# Post-traumatic stress disorder and its relation to the pandemic of the novel corona virus (COVID-19) in the bahraini society

**DOI:** 10.1192/j.eurpsy.2021.721

**Published:** 2021-08-13

**Authors:** A. Al Howaihi, A. Al Hamada

**Affiliations:** Psychiatry, Sulwan Psychiatric Center, building - flat - road -block, Bahrain

## Abstract

**Introduction:**

The world has given serious thought to epidemics, disasters and crises. One of the most important mental disorders that can be caused by epidemics, disasters and crises is the Post-Traumatic Stress Disorder (PTSD). The Coronavirus (COVID-19) pandemic is one of the most serious global health crises.

**Objectives:**

We deal with the appearance of symptoms of PTSD among Bahraini society as a consequence to COVID-19 pandemic. We aim to investigate two main aspects: PTSD and the correlation between the pandemic COVID-19 and the appearance of PTSD symptoms in the Bahraini society.

**Methods:**

This research was conducted based on the Davidson Trauma Scale (DTS), developed by Jonathan Davidson in 1995, according to the DSM-IV criteria.

**Results:**

The findings of our research concluded that the percentage of PTSD among a group of Bahraini society members following the outbreak of COVID-19 was 11.1%.

**Conclusions:**

conducting awareness campaigns as part of the media plan to combat COVID-19. Designing and applying treatment programs for PTSD for those in need. Allocating mental institutions from the public and private sectors to be used as rehabilitation centers for PTSD patients.

**Conflict of interest:**

No significant relationships.

